# Response to the COVID-19 Outbreak in Urban Settings in China

**DOI:** 10.21203/rs.3.rs-71833/v1

**Published:** 2020-09-09

**Authors:** Zhao Ni, Eli R. Lebowitz, Zhijie Zou, Honghong Wang, Huaping Liu, Roman Shrestha, Qing Zhang, Jianwei Hu, Shuying Yang, Lei Xu, Jianjun Wu, Frederick L. Altice

**Affiliations:** School of Medicine, Yale University, New Haven, CT, USA; School of Medicine, Yale University, New Haven, CT, USA; School of Health Sciences, Wuhan University, Hubei, China; Xiangya School of Nursing, Central South University, Hunan, China; School of Nursing, Peking Union Medical College, Beijing, China; School of Medicine, Yale University, New Haven, CT, USA; School of Health Sciences, Wuhan University, Hubei, China; College of Nursing, Xi’an Medical University, Xi’an, China; Hohhot Vocational College, Hohhot, China; School of Nursing, Fudan University, Shanghai, China; Gansu University of Chinese Medicine, Lanzhou, China; School of Medicine, Yale University, New Haven, CT, USA

**Keywords:** Coronavirus, COVID-19, anxiety, China, urban, health behavior, social life, global health

## Abstract

The COVID-19 outbreak in China was devastating, and spread throughout the country before being contained. Stringent physical distancing recommendations and shelter-in-place were first introduced in the hardest-hit provinces, and by March, these recommendations were uniform throughout the country. In the presence of an evolving and deadly pandemic, we sought to investigate the impact of this pandemic on individual well-being and prevention practices among Chinese urban residents. From March 2–11, 2020,4,607 individuals were recruited from 11 provinces with varying numbers of COVID-19 casers using the social networking app WeChat to complete a brief, anonymous, online survey. The analytical sample was restricted to 2,551 urban residents. Standardized scales measured generalized anxiety disorder (GAD), the primary outcome. Multiple logistic regression was conducted to identify correlates of GAD alongside assessment of community practices in response to the COVID-19 pandemic. We found that during the COVID-19 pandemic, recommended public health practices significantly (*p*<0.001) increased, including wearing facial mask, practicing physical distancing, handwashing, decreased public spitting, and going outside in urban communities. Overall, 40.3% of participants met screening criteria for GAD and 49.3%, 62.6%, and 55.4% reported that their work, social life, and family life were interrupted by anxious feelings, respectively. Independent correlates of having anxiety symptoms included being a healthcare provider (aOR=1.58, *p*<0.01), living in regions with a higher density of COVID-19 cases (aOR=2.13, *p*<0.01), having completed college (aOR=1.38, *p*=0.03), meeting screening criteria for depression (aOR=6.03,*p*<0.01) and poorer perceived health status (aOR=1.54,*p*<0.01). COVID-19 had a profound impact on the health of urban dwellers throughout China. Not only did they markedly increase their self- and community-protective behaviors, but they also experienced high levels of anxiety associated with a heightened vulnerability like depression, having poor perceived health, and the potential of increased exposure to COVID-19 such as living closer to the epicenter of the pandemic.

## 1. Introduction

The novel coronavirus disease 2019 (COVID-19) pandemic first rapidly spread throughout China, and by August 19^th^, 2020, it had manifested in 188 countries with 22,244,179 confirmed cases and 783,525 deaths worldwide.^1^ In the absence of effective vaccines or treatments, public health authorities have relied upon sheltering in place (self-quarantine at home), physical distancing in public settings, hand washing and wearing facial masks to prevent further spread.^2,3^ Without fully understanding its transmission, risk of progression, and widespread death from COVID-19, panic and even hysteria were common.^4^ The World Health Organization made public the COVID-19 outbreak in January 2020,^5^ and observed that the outbreaks were more severe in urban settings with a higher density of people.^6^ Consequently, Chinese residents increasingly complied with recommended containment measures that are necessary under this time of crisis, but those measures could disrupt their work and social and family life. Also, during the pandemic, many urban dwellers remained relatively segregated within their neighborhoods, and this negatively impacted their psychological well-being.^7,8^

Anxiety symptoms among urban dwellers dealing with a volatile COVID-19 pandemic, however, has not been broadly examined since it does not affect everyone equally. Over the past 18 years, various settings have reacted to new infectious diseases epidemics like SARS, MERS, and Ebola and, though none of these developed pandemic proportions, understanding factors that may undermine the health of the community are important for future public health disaster planning efforts. We, therefore, conducted a nationwide online survey of people in China to identify those factors associated with anxiety from COVID-19 and focused only on urban dwellers here, since they experienced COVID-19 differently than their non-urban counterparts.

## 2. Methods

### 2.1 Study design and participants

We conducted baseline, online survey with 4,607 participants living in China; two additional waves are underway. Participants inclusion criteria included: 1) ≥18 years old; 2) living in mainland China; 3) able to read Chinese; and 4) had access to WeChat (the largest social networking app in China). All recruited participants were asked to complete a baseline survey over ten days from March 2–11,2020. A total of 4,607 individuals from 11 provinces, with the varied impact of the COVID-19 pandemic, completed the online survey. The analytical sample was restricted to 2,551 urban residents who completed the enrollment survey. In this paper, the time point of COVID-19 outbreak refers to January 23^rd^, 2020, when Wuhan city was placed in quarantine. The study protocol was approved by the Institutional Review Board of Yale University and received ethical approval from Wuhan University.

### 2.2 Study procedures

**Figure 1:**
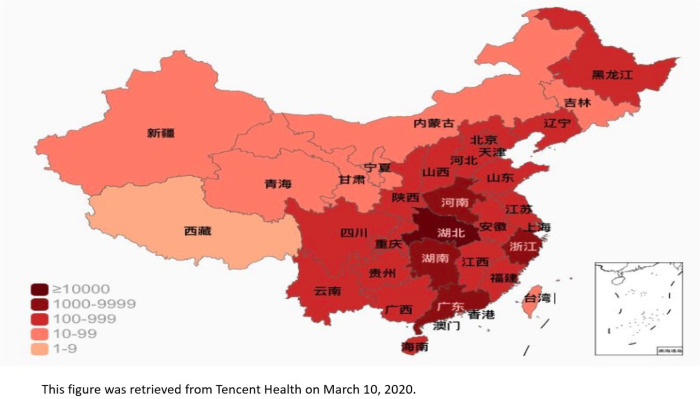
In this study, we used a modified snowball recruitment strategy where 11 participants (seeds) were recruited one each from 11 representative provinces in China. Eleven representative provinces were selected from mainland China based on two criteria: 1) being in one of mainland China’s six social-economic regions as classified by the National Bureau of Statistics of China: North (Beijing, Tianjin, Heibei, Shanxi, Inner Mongolia), Northeast (Liaoning, Jilin, Heilongjiang), East (Shanghai, Jiangsu, Zhejiang, Anhui, Fujian, Jiangxi, Shandong), Central South (Henan, Huibei, Hunan, Guangdong, Guangxi, Hainai), Southwest (Chongqing, Sichuan, Guizhou, Yunnan, Tibet), and Northwest (Shaanxi, Gansu, Qinghai, Ningxia, Xinjiang);9 and 2) COVID-19 severity as was categorized by China National Health CommissioniO (diagnosed COVID-19 cases≥ 10,000; 1,000–9,999; 100–999; ≤99) based on the percentage of provinces in each stratum in March 2020 Note: The designations employed and the presentation of the material on this map do not imply the expression of any opinion whatsoever on the part of Research Square concerning the legal status of any country, territory, city or area or of its authorities, or concerning the delimitation of its frontiers or boundaries. This map has been provided by the authors.

In this study, we used a modified snowball recruitment strategy where 11 participants (seeds) were recruited one each from 11 representative provinces in China. Eleven representative provinces were selected from mainland China based on two criteria: 1) being in one of mainland China’s six social-economic regions as classified by the National Bureau of Statistics of China: *North* (Beijing, Tianjin, Heibei, Shanxi, Inner Mongolia), *Northeast* (Liaoning, Jilin, Heilongjiang), *East* (Shanghai, Jiangsu, Zhejiang, Anhui, Fujian, Jiangxi, Shandong), *Central South* (Henan, Huibei, Hunan, Guangdong, Guangxi, Hainai), *Southwest* (Chongqing, Sichuan, Guizhou, Yunnan, Tibet), and *Northwest* (Shaanxi, Gansu, Qinghai, Ningxia, Xinjiang);^9^ and 2) COVID-19 severity as was categorized by China National Health Commission^10^ (diagnosed COVID-19 cases≥ 10,000; 1,000–9,999; 100–999; ≤99) based on the percentage of provinces in each stratum in March 2020 (Figure 1). Using these criteria, we selected the following 11 representative provinces: Beijing, Inner Mongolia, Heilongjiang, Shandong, Henan, Hubei, Hunan, Guizhou, Shaanxi, Gansu, and Xinjiang. Seeds were recruited using convenience sampling method.

To address the impact of the COVID-19 pandemic, the survey was developed, and pilot tested using methods that have been described elsewhere.^11^ In brief, standardized scales were used, and responses to COVID-19 were created. After drafting candidate questions, ten experts in the field took the survey and provided feedback to refine the survey. The revised survey was then designed on Questionnaire Star (https://www.wjx.cn/), a professional platform for online surveys,^12^ and a web link, and a QR code was generated. We then pilot-tested the survey with 32 individuals who accessed the survey from a weblink or QR code and sought feedback. Using feedback, we finalized the electronic survey and applied the web-based sampling method to recruit participants after identifying the seed in each province.

The selected 11 seed participants completed the survey and then distributed a flyer that contained recruitment information, quick response (QR) code, and a link to the online survey among their social network. The distribution of the flyer occurred through WeChat Moments (“Peng You Quan” in Chinese) or their WeChat groups (“Wei Xin Qun” in Chinese). Interested individuals who clicked on the link were directed to an eligibility screener. Each eligible participant voluntarily completed an online consent form by acknowledging that they understood the purpose, risks, and benefits of the study prior to completing the survey. On average, participants took 12 minutes to complete the anonymous online survey. The questionnaire was available in both English and Chinese languages and was translated and back-translated to ensure culture meaning.^13^

### 2.3 Study measures

Sociodemographic characteristics included age, sex, educational level, income, health, employment, and marital status. Income was stratified based on the relationship to the national levels.

Traveling history in the past 30 days included whether they had traveled after the COVID-19 outbreak, and whether they were put in quarantine. Living environment was based on with whom they lived, and the region where they lived, stratified by the density of COVID-19 cases, with Hubei province being the highest. We also measured where participants accessed information pertaining to COVID-19 and what measures that their communities had taken to control COVID-19.

Participants’ self-perceived health status were measured by the question “How is your current health status?” with a response of “Very good”, “Good”, “Fair”, “Poor”, and “Very poor”. These answers were dichotomized into “Good” (“Very good” + “Good”), and “Not good” (“Fair” + “Poor” + “Very poor”). In addition, we assessed the frequency of the following health-related behaviors, before and after the COVID-19 outbreak, which included wearing face masks, practicing physical distancing, washing hands, spitting, and showering. The questions related to each construct are included in Table 2.

The primary outcome was the presence of anxiety symptoms severity, which was measured by the Generalized Anxiety Disorder 7-item (GAD-7) scale, which has good reliability, sensitivity, and specificity for measuring anxiety in Chinese populations.^14^ Generalized anxiety disorder (GAD) cut-offs for mild, moderate, and severe symptoms including scores of 5–9,10–14, and >15, respectively. Other screening for mental illness included assessment of obsessive-compulsive symptoms using the Obsessive-Compulsive Inventory^15^ and depression using the Patient Health Questionnaire-2.^16^

### 2.4 Statistical analyses

All data analyses were performed using SAS 9.4 (SAS Institute, Cary, North Carolina, United States). Data were presented using frequencies and means. Chi-square test was used to compare the behaviors of wearing face masks and practicing physical distancing before and after the COVID-19. Student’s t-test was used to examine differences in hand washing, spitting, going outside, and showering, before and after the outbreak. Logistic regression was used to examine the association between potential explanatory variables and the presence of anxiety. Anxiety was dichotomized for values >4, which is associated with the presence of anxiety symptoms. Any variable significant at *p*<0.10 in bivariate analyses were then entered into the multivariate logistic regression model to determine the odds ratio and 95% confidence intervals for the final model. An additional analysis (Supplementary Data) for moderate to severe anxiety symptoms (cut-off >9) was also conducted.

## 3. Results

### 3.1. Participant characteristics

**Figure 2:**
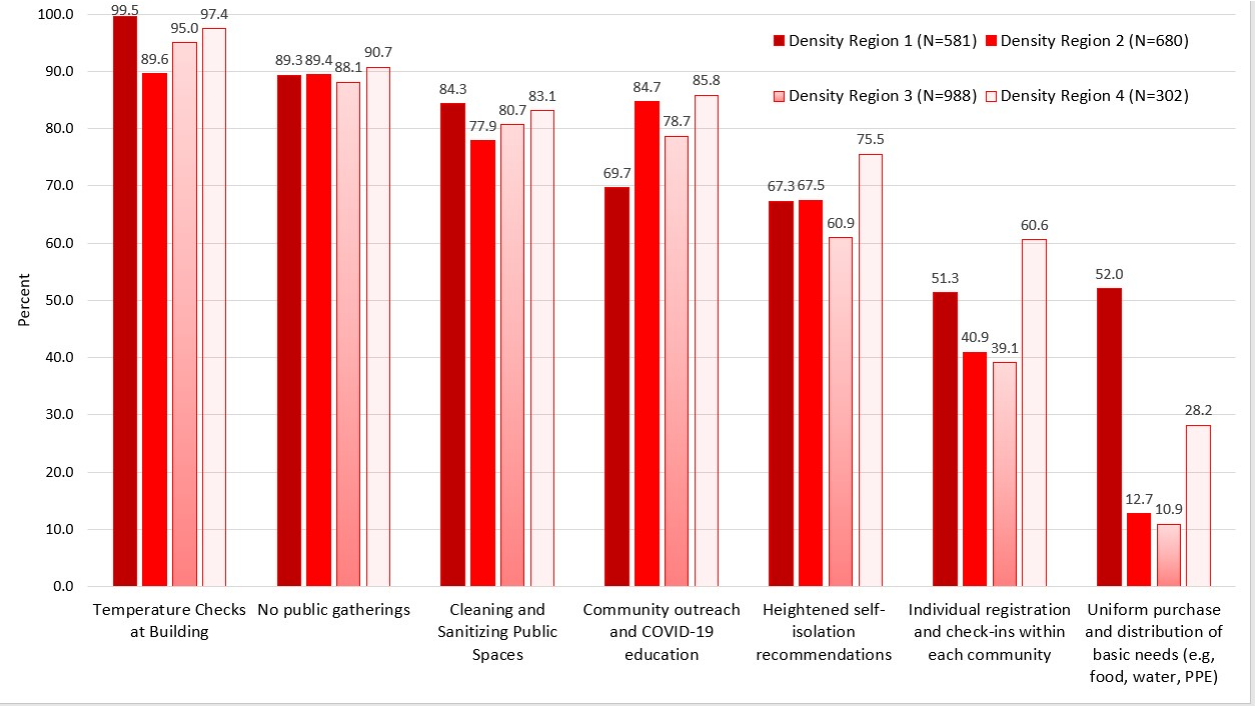
Participants were from regions with different density of COVID-19 cases, 22.8% of them were from the epicenter - Hubei province. Nearly half of the participants were married (47.4%). Most participants reported that they didn’t travel (95.5%) after the COVID-19 outbreak, and most communities (93.4%) had taken strict measures to control COVID-19. Overall, the top three commonly used preventative measures in Chinese urban areas were: controlling the entry and exit of people by checking their body temperature, banning gatherings in the community, and cleaning and sanitizing communal spaces (Figure 2).

Most participants (Table 1) were female (68.9%), in their 30s (31.3±11.9), completed a college degree (89.5%), and perceived themselves to be in good health status (74.8%). Nearly 34% of the participants have an annual income of greater than ¥ 60,000 (12 times greater than the international poverty threshold; equivalent to 8,571 USD), and 16.0% of the participants were healthcare providers. Nearly all (93.1 %) participants were living with families and remained in one city during the 30 days prior to the study. Participants were from regions with different density of COVID-19 cases, 22.8% of them were from the epicenter - Hubei province. Nearly half of the participants were married (47.4%). Most participants reported that they didn’t travel (95.5%) after the COVID-19 outbreak, and most communities (93.4%) had taken strict measures to control COVID-19. Overall, the top three commonly used preventative measures in Chinese urban areas were: controlling the entry and exit of people by checking their body temperature, banning gatherings in the community, and cleaning and sanitizing communal spaces (Figure 2).

### 3.2. Health-related behaviors before and after the COVID-19 outbreak

The number of participants who wore face masks and practiced physical distancing, and the frequency of hand washing increased significantly after the COVID-19 outbreak (*p*<0.001). The rate of spitting in public places and going outside of one’s home decreased significantly (*p*<0.001; Table 2).

### 3.3. Correlates of having generalized anxiety disorder

Several independent correlates were associated with having mild, moderate, and severe anxiety symptoms, including poor perceived health status (aOR=1.54, *p*<0.01), being a healthcare provider (aOR=1.58, *p*<0.01), received a college degree or above (aOR=1.38, *p*=0.03), living in Hubei (aOR=2.13, *p*<0.01), and meeting screening criteria for depression (aOR=6.03, *p*<0.01; Table 3).

### 3.4 Correlates of moderate to severe generalized anxiety disorder

As shown in Table 4 in the supplementary appendix, poor self-perceived health status (aOR=1.73, *p*<0.01), higher frequency of washing hands (aOR=1.02, *p*=0.03), living in Hubei (aOR=2.85, *p*<0.01), and meeting screening criteria for depression (aOR=24.20, *p*<0.01) were independently associated with moderate and severe anxiety symptoms.

## 4. Discussion

The unprecedented COVID-19 pandemic has raised significant public health concerns and has an extended impact on the psychological well-being of society, especially in urban areas most profoundly impacted by the disease. The COVID-19 epidemic unleashed a rapid and cataclysmic response by society, in which we report the profound protective response to the COVID-19 outbreak. In response to government guidance and clear messaging, frequency of hand washing and physical distancing practices increased, while venturing outside in crowded urban spaces or spitting in public places decreased. Though public spitting is unlawful in some Chinese cities like Beijing,^17^ Hangzhou,^18^ and Tianjin,^19^ it remains legal and practiced elsewhere; but during COVID-19, such practices markedly reduced. On May 15,2020, the Chinese Government of Shanxi province passed China’s first provincial law prohibiting spitting in public places, which aimed to change uncivilized behaviors and prevent the spread of infectious diseases.^20,21^ Unlike physical distancing and handwashing that were widely recommended by public health authorities’ sources, public spitting messages were mostly from non-official online sources. Another explanation for a decrease in this behavior is that people remained inside more and such public spitting opportunities were less. These findings do not appear to be driven by social desirability response since other hygienic measures that were not suggested in governmental and public sources, like showering, were not impacted.

Anxiety levels were high in this large sample. Surveys from multiple countries, including China,^22^ Germany,^23^ Italy,^24^ Saudi Arabia,^25^ and Turkey^26^ have shown that the prevalence of anxiety increased significantly with the global escalation of the COVID-19 pandemic. For example, prior to the COVID-19 outbreak, the prevalence of anxiety among a national sample of 38,294 Chinese urban dwellers was 5.3%,^27^ and in a post-COVID survey of 7,236 Chinese citizens,^22^ the prevalence rose to 35.1 *%* using the same GAD screening instrument. Our study had a similar prevalence to the other, but we identified more factors that were correlated with GAD. Unlike the other survey that found younger age (<35 years) and time spent (>3 hours daily) focusing on COVID-19, our assessment of urban dwellers found that GAD was correlated with being a healthcare worker, living in region more profoundly impacted by COVID-19, having poorer self-perceived health status, having a college education and having moderate to severe depression.

Findings from our urban study, combined with those from both urban and non-urban dwellers, underscores the importance of providing support to a large number of people impacted by a new and evolving epidemic. Our findings, however, provide important insights into how to focus such intervention efforts to provide trauma-informed care. For example, healthcare workers, which have been identified elsewhere to experience exceptional levels of stress, should be targeted for screening and intervention. Additionally, those with lower self-perceived health should be targeted. Many such individuals may potentially have co-morbid conditions that increase their likelihood of experiencing more severe COVID-19 disease if they become infected.^28,29^ This is especially true since they may perceive they are unable to access needed healthcare services since during the pandemic, only essential medical visits were allowed, leaving them without support to self-manage their medical conditions. While patients with depression may also experience anxiety symptoms, in our survey, these variables were not collinear, but suggests that such patients have a lower psychological reserve to deal with stress and experienced heightened anxiety symptoms. This finding is born out in our additional analysis that shows depression is highly correlated with moderate to severe anxiety symptoms.

In the initial stage of responding to COVID-19, most healthcare facilities in the outbreak regions shuttered their doors to patients, except for those with urgent needs. Consequently, care was transitioned to tele-health. One potential implication from this survey is that healthcare providers, when providing tele-health to patients with chronic diseases that may heighten risk for more severe consequences of COVID-19, and even those with depression, should screen such patients for GAD and provide supportive counseling, which can effectively be done using tele-health.^30^

As pandemics evolve, unscientific ideas may proliferate about how infections can be prevented, treated and cured. In the early stage of COVID-19, rumors of several effective treatments were touted to suppress COVID-19 from unsubstantiated online sources, which in turn generated the public anxiety because everyone wanted the treatments, yet they were unavailable for purchase.^31^ Providing accurate health information guided by science is therefore important to mitigate excess anxiety during the pandemic. Unsubstantiated rumors have been found to provoke anxiety and exacerbate mental health before SARS, avian flu, and swine flu epidemics.^32–34^ In times of crisis, it is even more important to ensure information is accurate and scientifically grounded to ensure that people feel safe. In the case of COVID-19, considerable uncertainty existed and in an evolving crisis, conspiracy theories and hyperbole abound which, in turn, perpetuates anxiety.^35^ Health information, however, often comes from multiple sources, but should be derived from someone who is respected, has authority and trusted by society.

During an infectious pandemic that requires physical distancing, mobile technology may be crucial as a conduit of accurate (and sometimes inaccurate) information.^36,37^ Such information is more powerful, however, when collaborative learning is used and people can teach each other as long as an expert is there to guide discussion.^38^ Collaborative learning in communities, defined as integrating meaningful community engagement with education, instruction, and reflection to promote the capacity of individuals to take collective actions to improve the quality of life, is a key method considered by many international and national bodies to prepare for, respond to, and recover from emergency situations.^39,40^ Mobile technology-based interventions (e.g., telemedicine) could easily be repurposed to promote community learning not only as a dissemination method of accurate information, but also to address anxiety, maintain social connectivity while physically distancing, mobilize resources, and support community-based networks of people in need.^41^ For instance, a tele-health visit using video or telephone from local clinicians could screen, motivate and treat patients and families. Even when stigma about mental illness is common, as it is in China,^27^ brief motivational enhancement techniques can be deployed as part of trauma-informed care that can be done routinely without making a diagnosis. Building such interventions and messages in public forums and giving people an opportunity to discuss how the pandemic is affecting them can provide an open opportunity for assistance. This would be especially crucial in some regions of mainland China where it might be considered “abnormal” or a shameful to seek treatment for anxiety. Such individual or public messaging to provide trauma-informed care to individuals with anxiety would minimally include examples to support self-regulation of stressors, prioritize healthy relationships, explain why health restrictions are being made that otherwise limit routine daily activities, visualize what to expect within reason of what is known, and reframe behaviors to account for people not being at their best during times of crisis.^42^

It is no surprise that urban dwellers living closest to the epicenter and with the high density of COVID-19 cases (e.g., Hubei) experienced the most anxiety, relative to those in less dense COVID-19 cases. These individuals had the most uncertainty as they were impacted first and had the least amount of accurate information. Such individuals might have also perceived themselves at highest risk, which is similar to our finding that healthcare workers, also at substantial risk, experienced heightened anxiety symptoms. Of note, healthcare workers had an increased association of experiencing mild anxiety symptoms, but not moderate or severe anxiety symptoms. One might expect that such individuals would have the most severe anxiety symptoms because they are at the highest risk for COVID-19 combined with extreme workloads during a heightened crisis management scenario where personal protective equipment and testing were inadequate.^43^ One potential explanation is that healthcare workers self-manage life and death situations on a daily basis and have established functional coping mechanisms. Alternatively, data from Wuhan suggested that over half of healthcare workers accessed support services, which may have helped them better deal with anxiety-provoking stressors.^44^ Last, the healthcare workers in this survey may not have been those providing the most direct patient care and therefore did not experience the highest levels of anxiety.

Though this large survey assessing responses and anxiety symptoms across a large number of regions of China had many important and new findings, it is without limitations. First, convenience sampling using WeChat does not make this a fully representative sample and restrict generalizability. Second, though markedly higher levels of generalized anxiety disorders were reported relative to the general population before COVID-19, we could not infer that COVID-19 was causative due to the cross-sectional nature of the survey. Last, some factors that may have contributed to anxiety symptoms may not have been measured, like time spent online seeking COVID-related information or various types of coping mechanism. Future research should more comprehensively study the possible negative psychological consequences of various countermeasures to find out the best solution. Finally, this study compared anxiety levels from before the outbreak to March 2020 but did not assess changes in anxiety levels over the entire period of the pandemic. More research should be conducted to examine changes in mental health outcomes over the entire pandemic period.

## 5. Conclusion

COVID-19 has had a profound impact on China initially and continues to do so globally. In China, urban residents markedly changed their health behaviors in response to the evolving epidemic. These urban dwellers also experienced profound levels of anxiety, especially in settings closest most profoundly impacted by the epidemic and in those most vulnerable like healthcare workers and those with poor perceived health, including those with depression. Much has been learned from prior epidemics to guide a trauma-informed response, but when physical distancing practices are imposed, innovations in reaching screening, motivating and treating such individuals at increased risk for anxiety are urgently needed. Technology-based interventions like online collaborative learning environments and tele-health can be used to solve such obstacles to service delivery. Such lessons can be useful as new settings become susceptible to COVID-19 and as secondary outbreaks emerge before an effective vaccine is made widely available.

## Supplementary Material

Supplement

## Figures and Tables

**Table 1.** Characteristics of participants (N=2,551)

Variables	Sample	
	Frequency	%
**Age** (years), mean (SD)	31.3	11.9
**Sex**		
Female	1758	68.9
Male	793	31.1
**Educational level**		
College degree or above	2284	89.5
High school or less	267	10.5
**Marital status**		
Single	1270	49.8
Married	1210	47.4
Divorced	58	2.3
Lost spouse	13	0.5
**Self-perceived health status**		
Not good	642	25.2
Good	1909	74.8
**Job**		
No job	86	3.4
Retired	87	3.4
Government employee	88	3.5
Healthcare provider	408	16.0
Company employee	395	15.5
Teacher	210	8.2
Students	956	37.5
Self-employed	203	8.0
Farmer	15	0.6
**Annual income**		
≥ 12 times of the international poverty threshold	862	33.8
9 – 12 times	502	19.7
6 – 9 times	583	22.9
< 6 times	604	23.7
**From regions with different density of COVID-19 cases (March 2020)**		
Hubei (≥10000 cases)	581	22.8
2^nd^ highest region (1000–9999 cases)	680	26.7
3^rd^ highest region (100–999 cases)	988	38.7
Low density region (1–99 cases)	302	11.8
**Living alone**		
Yes	177	6.9
No	2374	93.1
**Measures taken to control COVID-19 in your community**		
Very strict	1249	49.0
Strict	1133	44.4
Fair	160	6.3
Loose	9	0.3
**Traveled after the COVID-19 outbreak**		
Yes	116	4.6
No	2435	95.5
**In quarantine**		
Yes	219	8.6
No	2332	91.4
**Reasons of being put in quarantine**		
Diagnosed with COVID-19	2	0.1
Has symptoms of COVID-19	2	0.1
Had been in contact with COVID-19	16	0.6
Returning hometown from other communities where there were COVID-19 patients	199	7.8
**Where people learned about updated information of the COVID-19**		
APP (WeChat, QQ, NetEase, and etc.)	2405	94.3
Website	1713	67.2
Radio	659	25.8
TV	1817	71.2
Journal	231	9.1
Family or relatives	1293	50.7
Friends	1083	42.5
Colleagues	723	28.3
**Depression**		
Yes	381	14.9
No	2170	85.1
**Generalized Anxiety Disorder**		
Mild	832	32.6
Moderate	150	5.9
Severe	46	1.8
Any	1028	40.3
No consistent symptom	1523	59.7
**Work / schoolwork has been disrupted**		
Yes	1258	49.3
No	1293	50.7
**Social life has been disrupted**		
Yes	1597	62.6
No	954	37.4
**Family life / home responsibilities have been disrupted**		
Yes	1414	55.4
No	1137	44.6
**Presence of obsessive-compulsive disorder**		
Yes	232	9.1
No	2319	90.9

**Table 2.** Comparing health-related behaviors before and after the COVID-19 outbreak

Health-related behaviors	Description	Before the COVID-19 outbreak		After the COVID-19 outbreak		*P* value
**Wearing face masks**	When people had a cold or fever, they would always wear a face mask if they went outside of their house or apartment	**Sample** **(N=2,551)**		**Sample** **(N=2,551)**		<0.001*
		**Frequency**	**%**	**Frequency**	**%**	
Yes		1156	45.3	2543	99.7	
No ^‡^		1395	54.7	8	0.3	
**Practicing physical distancing**	When people used public transportation or were inside a building and noticed that someone else seemed to have a cold or a fever (coughing, sneezing, etc.), they would change their location or try to get away from others					<0.001*
Yes		1830	71.7	2481	97.3	
No		721	28.3	70	2.7	
		**Mean**	***SD***	**Mean**	***SD***	
**Washing hands**	The average number of times that people washed their hands daily with soap (or hand sanitizer) and running water	5.0	4.6	7.7	7.0	<0.001*
**Spiting**	The average number of times that people spat on the ground weekly in public places	0.4	1.7	0.1	0.9	<0.001*
**Going outside**	The average number of times that people went outside weekly of their house or apartment	6.1	5.2	2.2	3.0	<0.001*
**Taking shower**	The average number of times that people took a shower weekly	3.7	2.2	3.7	2.4	0.45

‡ 423 participants, who reported that they sometimes wore a face mask, sometimes didn’t, were categorized into this group.

* Variables that have been significant at 0.05 level.

**Table 3.** Bivariate and Multivariate Correlates of Having Symptoms of Generalized Anxiety Disorder (N=2,551)

Variable	N	Bivariate Associations			Multivariate Analysis		
		*OR*	95% *CI*	*P*-value	*aOR*	95% *CI*	*P*-value
**Age** (years; continuous)	2551	1.0	0.99, 1.00	0.22			
**Sex**	2551						
Female	1758	1.05	0.89, 1.25	0.57			
Male (ref)	793						
**Educational level**	2551						
College degree or above	2284	1.35	1.03, 1.76	0.03*	1.38	1.03, 1.86	0.03**
High school or below (ref)	267						
**Marital status**	2551						
Married ^‡^	1281	1.13	0.97, 1.32	0.13			
Single (ref)	1270						
**Self-perceived health status**	2551						
Not good	642	1.69	1.41, 2.02	<0.01*	1.54	1.27, 1.87	<0.01**
Good (ref)	1909						
**Healthcare worker**	2551						
Yes	408	1.56	1.26, 1.93	<0.01*	1.58	1.23, 2.02	<0.01**
No (ref)	2143						
**Annual income**	2551						
≥ 12 times of the international poverty threshold	862	1.21	0.98, 1.50	0.08*	0.98	0.76, 1.26	0.89
9 – 12 times	502	1.29	1.01, 1.64	0.04*	1.16	0.89, 1.51	0.29
6 – 9 times	583	1.27	1.01, 1.61	0.04*	1.14	0.88, 1.47	0.31
< 6 times (ref)	604						
**From regions with different density of COVID-19 cases**	2551						
Hubei (≥10,000 cases)	581	2.03	1.52, 2.71	<0.01*	2.13	1.54, 2.95	<0.01**
2^nd^ highest region (1000–9999 cases)	680	1.12	0.84, 1.49	0.44	1.11	0.81, 1.52	0.51
3^rd^ highest region (100–999 cases)	988	1.11	0.85, 1.45	0.45	1.18	0.88, 1.59	0.27
Low density region (1–99 cases; ref)	302						
**Living alone**	2551						
Yes	177	1.37	1.01, 1.86	0.04*	1.02	0.73, 1.44	0.89
No (ref)	2374						
**Measures taken to control COVID-19 in your community**	2551						
Very strict	1249	1.24	0.31, 5.00	0.76			
Strict	1133	1.44	0.36, 5.78	0.61			
Fairly strict	160	1.64	0.40, 6.77	0.50			
Loose (ref)	9						
**Traveled after the COVID-19 outbreak**							
Yes	116	1.46	1.00, 2.12	0.05*	1.34	0.89, 2.03	0.16
No (ref)	2435						
**In quarantine**	2551						
Yes	219	1.38	1.04, 1.82	0.02*	1.31	0.97, 1.77	0.08
No (ref)	2332						
**Depression**	2551						
Yes	381	6.29	4.88, 8.09	<0.01*	6.03	4.66, 7.81	<0.01**
No (ref)	2170						
**Wearing face masks** ^A^	2551						
Yes	2543	0.67	0.17, 2.70	0.58			
No (ref)	8						
**Wearing face masks** ^B^	2551						
Yes	1156	0.80	0.68, 0.94	<0.01*	0.89	0.75, 1.07	0.21
No (ref)	1395						
**Practicing social distancing** ^A^	2551						
Yes	2481	1.08	0.66, 1.75	0.77			
No (ref)	70						
**Practicing social distancing** ^B^	2551						
Yes	1830	0.94	0.79, 1.12	0.50			
No (ref)	721						
**Washing hands** ^A^ (number; continuous)	2551	1.01	1.00, 1.02	0.05*	1.01	0.99, 1.02	0.34
**Washing hands** ^B^ (number; continuous)	2551	1.01	1.00, 1.03	0.21			
**Spitting** ^A^ (number; continuous)	2551	1.08	0.99, 1.18	0.11			
**Spitting** ^B^ (number; continuous)	2551	1.02	0.98, 1.07	0.37			
**Going outside** ^A^ (number; continuous)	2551	1.00	0.98, 1.03	0.77			
**Going outside** ^B^ (number; continuous)	2551	1.02	1.00, 1.03	0.04*	1.01	0.99, 1.03	0.39
**Taking shower** ^A^ (number; continuous)	2551	1.04	1.01, 1.08	0.01*	0.99	0.93, 1.06	0.78
**Taking shower** ^B^ (number; continuous)	2551	1.05	1.01, 1.09	<0.01*	1.03	0.96, 1.11	0.38

*OR* odds ratio, *aOR* adjusted odds ratio *CI* confidence interval, *ref* reference group.

* In bivariate logistic regression models, those variables whose P-value is less than 0.1 was included in the multiple logistic regression.

** Variables that have been significant at 0.05 level in multiple logistic regression model.

‡ Participants who divorced or lost spouse were categorized into the categorize of Married.

A Health-related behavior after the COVID-19 outbreak.

B Health-related behavior before the COVID-19 outbreak.
