# Supplementary material for: Response to the COVID-19 Outbreak in Urban Settings in China
Source: Res Sq. 2020 Sep 9:rs.3.rs-71833. Preprint. [Version 1] doi: 10.21203/rs.3.rs-71833/v1 (PMC7491581; doi:10.21203/rs.3.rs-71833/v1)
Supplement: Supplement [file Supplementalappendix.docx]

**Supplemental appendix**

**Table 4. Bivariate and Multivariate Correlates of Having Moderate to Severe Symptoms of Generalized Anxiety Disorder (N=2,551)**

| **Variable** | **N** | **Bivariate Associations** | | |  | **Multivariate Analysis** | | |
| --- | --- | --- | --- | --- | --- | --- | --- | --- |
|  |  | ***OR*** | **95% *CI*** | ***P*-value** |  | ***aOR*** | **95% *CI*** | ***P*-value** |
| **Age** (years; continuous) | 2551 | 0.99 | 0.98, 1.01 | 0.26 |  |  |  |  |
| **Sex** | 2551 |  |  |  |  |  |  |  |
| Female | 1758 | 1.05 | 0.89, 1.25 | 0.05* |  | 0.87 | 0.61, 1.25 | 0.46 |
| Male (ref) | 793 |  |  |  |  |  |  |  |
| **Educational level** | 2551 |  |  |  |  |  |  |  |
| College degree or above | 2284 | 0.83 | 0.53, 1.29 | 0.40 |  |  |  |  |
| High school or less (ref) | 267 |  |  |  |  |  |  |  |
| **Marital status** | 2551 |  |  |  |  |  |  |  |
| Married ^‡^ | 1281 | 1.04 | 0.77, 1.39 | 0.81 |  |  |  |  |
| Single (ref) | 1270 |  |  |  |  |  |  |  |
| **Self-perceived health status** | 2551 |  |  |  |  |  |  |  |
| Not good | 642 | 2.25 | 1.67, 3.04 | <0.01* |  | 1.73 | 1.20, 2.48 | <0.01** |
| Good (ref) | 1909 |  |  |  |  |  |  |  |
| **Job** | 2551 |  |  |  |  |  |  |  |
| Healthcare provider | 408 | 0.79 | 0.52, 1.21 | 0.28 |  |  |  |  |
| Not healthcare provider (ref) | 2143 |  |  |  |  |  |  |  |
| **Annual income** | 2551 |  |  |  |  |  |  |  |
| ≥ 12 times of the international poverty threshold | 862 | 1.11 | 0.75, 1.64 | 0.61 |  |  |  |  |
| 9 – 12 times | 502 | 0.95 | 0.60, 1.51 | 0.84 |  |  |  |  |
| 6 – 9 times | 583 | 1.14 | 0.75, 1.75 | 0.54 |  |  |  |  |
| < 6 times (ref) | 604 |  |  |  |  |  |  |  |
| **From regions with different density of COVID-19 cases** | 2551 |  |  |  |  |  |  |  |
| Hubei (≥10,000 cases) | 581 | 2.52 | 1.48, 4.29 | <0.01* |  | 2.85 | 1.52, 5.34 | <0.01** |
| 2^nd^ highest region (1000-9999 cases) | 680 | 1.23 | 0.70, 2.14 | 0.48 |  | 1.43 | 0.76, 2.71 | 0.27 |
| 3^rd^ highest region (100-999 cases) | 988 | 0.82 | 0.47, 1.44 | 0.49 |  | 1.06 | 0.56, 1.99 | 0.86 |
| Low density region (1-99 cases; ref) | 302 |  |  |  |  |  |  |  |
| **Living alone** | 2551 |  |  |  |  |  |  |  |
| Yes | 177 | 2.12 | 1.35, 3.33 | <0.01* |  | 1.71 | 0.99, 2.98 | 0.06 |
| No (ref) | 2374 |  |  |  |  |  |  |  |
| **Measures taken to control COVID-19 in your community** | 2551 |  |  |  |  |  |  |  |
| Very strict | 1249 | 0.70 | 0.09, 5.68 | 0.74 |  |  |  |  |
| Strict | 1133 | 0.53 | 0.07, 4.27 | 0.55 |  |  |  |  |
| Fairly strict | 160 | 1.41 | 0.17, 11.81 | 0.75 |  |  |  |  |
| Loose (ref) | 9 |  |  |  |  |  |  |  |
| **Traveled after the COVID-19 outbreak** |  |  |  |  |  |  |  |  |
| Yes | 116 | 1.55 | 0.86, 2.82 | 0.15 |  |  |  |  |
| No (ref) | 2435 |  |  |  |  |  |  |  |
| **In quarantine** | 2551 |  |  |  |  |  |  |  |
| Yes | 219 | 1.55 | 0.98, 2.43 | 0.06* |  | 1.30 | 0.75, 2.26 | 0.35 |
| No (ref) | 2332 |  |  |  |  |  |  |  |
| **Depression** | 2551 |  |  |  |  |  |  |  |
| Yes | 381 | 25.53 | 18.06, 36.09 | <0.01* |  | 24.20 | 16.88, 34.69 | <0.01** |
| No (ref) | 2170 |  |  |  |  |  |  |  |
| **Wearing face masks ^A^** | 2551 |  |  |  |  |  |  |  |
| Yes | 2543 | 0.58 | 0.07, 4.75 | 0.61 |  |  |  |  |
| No (ref) | 8 |  |  |  |  |  |  |  |
| **Wearing face masks ^B^** | 2551 |  |  |  |  |  |  |  |
| Yes | 1156 | 0.78 | 0.58, 1.05 | 0.11 |  |  |  |  |
| No (ref) | 1395 |  |  |  |  |  |  |  |
| **Practicing social distancing ^A^** | 2551 |  |  |  |  |  |  |  |
| Yes | 2481 | 1.08 | 0.43, 2.73 | 0.86 |  |  |  |  |
| No (ref) | 70 |  |  |  |  |  |  |  |
| **Practicing social distancing ^B^** | 2551 |  |  |  |  |  |  |  |
| Yes | 1830 | 0.72 | 0.53, 0.98 | 0.04* |  | 0.84 | 0.58, 1.21 | 0.35 |
| No (ref) | 721 |  |  |  |  |  |  |  |
| **Washing hands ^A^** (number; continuous) | 2551 | 1.02 | 1.00, 1.03 | 0.09* |  | 1.02 | 1.00, 1.05 | 0.03** |
| **Washing hands ^B^** (number; continuous) | 2551 | 1.01 | 0.98, 1.04 | 0.69 |  |  |  |  |
| **Spitting ^A^** (number; continuous) | 2551 | 1.15 | 1.04, 1.28 | <0.01* |  | 1.12 | 0.98, 1.29 | 0.09 |
| **Spitting ^B^** (number; continuous) | 2551 | 1.05 | 0.99, 1.12 | 0.13 |  |  |  |  |
| **Going outside ^A^** (number; continuous) | 2551 | 0.96 | 0.90, 1.01 | 0.11 |  |  |  |  |
| **Going outside ^B^** (number; continuous) | 2551 | 1.02 | 0.99, 1.04 | 0.22 |  |  |  |  |
| **Taking shower ^A^** (number; continuous) | 2551 | 1.08 | 1.02, 1.14 | <0.01* |  | 1.09 | 0.97, 1.23 | 0.16 |
| **Taking shower ^B^** (number; continuous) | 2551 | 1.06 | 1.00, 1.13 | 0.05* |  | 0.94 | 0.83, 1.07 | 0.37 |

*OR* odds ratio, *aOR* adjusted odds ratio *CI* confidence interval, *ref* reference group.

* In bivariate logistic regression models, those variables whose *P*-value is less than 0.1 was included in the multiple logistic regression.

** Variables that have been significant at 0.05 level in multiple logistic regression model.

^‡^ Participants who divorced or lost spouse were categorized into the categorize of Married.

**^A^** Health-related behavior after the COVID-19 outbreak.

**^B^** Health-related behavior before the COVID-19 outbreak.
